# *Curvularia lunata* causing orbital cellulitis in a diabetic patient: An old fungus in a new territory

**DOI:** 10.18502/cmm.6.1.2510

**Published:** 2020

**Authors:** Himanshu Narula, Suneeta Meena, Sweta Jha, Neelam Kaistha, Monika Pathania, Pratima Gupta

**Affiliations:** 1 Department of Microbiology, All India Institute of Medical Sciences, Rishikesh; 2 Department of Medicine, All India Institute of Medical Sciences, Rishikesh; 3 Department of Microbiology, All India Institute of Medical Sciences, Rishikesh

**Keywords:** Curvularia, Diabetes mellitus, Fungal rhinosinusitis, Orbital Cellulitis

## Abstract

**Background and Purpose:**

Rhinocerebral mycosis is a rapidly invasive infection in diabetic patients with an unfavorable course. Herein, we report a rare case of orbital cellulitis caused by *Curvularia lunata *following fungal rhinosinusitis in a diabetic male patient.

**Case report:**

A 35-year-old male with uncontrolled diabetes presented to the emergency department of our center with high-grade fever accompanied by chills and rigors, severe diffuse headache, and projectile vomiting with swelling and loss of vision in the right eye. The tissue sample from surgical debridement showed pigmented hyphae; in addition, *Curvularia lunata* was isolated in culture. Imaging was indicative of orbital extension. Therefore, the patient was diagnosed with fungal rhinosinusitis with orbital cellulitis. The patient was subjected to extensive surgical debridement, along with antifungals. Rhinosinusitis resolved; however, the loss of vision was irreversible.

**Conclusion:**

Orbital cellulitis is a very rare but life-threatening complication of fungal rhinosinusitis. Very few cases of orbital cellulitis following fungal rhinosinusitis have been reported in the literature. Early and prompt diagnosis can save the life of a patient.

## Introduction

*Curvularia* infections are relatively uncommon in humans despite the ubiquitous nature of these dematiaceous fungi in the environment. *Curvularia lunata* has been reported as the etiological agent of keratomycosis, sinusitis, onychomycosis, eumycetoma, pneumonia, endocarditis, dialysis-associated peritonitis, cerebral abscess, and disseminated fungal infections. This species has also been reported to account for ocular infections, such as conjunctivitis, dacryocystitis, and endophthalmitis [1-4]. 

*Curvularia lunata* has been rarely identified as a causative pathogen of invasive infections in diabetic patients causing vision loss. *Curvularia lunata* and *C. geniculata* are the most common species known to cause ocular infections. Nevertheless, there are also case reports on fungal keratitis caused by the rare species of *C. senegalensis *[1]. We report a case of orbital cellulitis caused by *C. lunata* in a patient with incidentally diagnosed type II diabetes mellitus.

## Case report

A 35-year-old male businessman presented to the emergency department of our center with the chief complaints of fever for the last two weeks, severe diffuse headache and vomiting for the past one week, and swelling and loss of vision in the right eye for the past two days. Fever was high-grade and intermittent with chills and rigors and was not relieved by over-the-counter medication, namely diclofenac 50 mg, used daily for 7 days. After one week of febrile illness, the patient developed a severe diffuse headache and nonprojectile vomiting for which he consulted a local practitioner and was admitted and started on empirical intravenous antibiotics (i.e., piperacillin/tazobactam). Two days after admission, he developed pain and swelling accompanied by loss of vision in the right eye, for which the patient was referred to our center. There was no significant history of smoking, alcohol consumption, medical illness or trauma to the face or eye in the past.

Physical examination revealed a temperature of 100.4 F and blood pressure of 101/67 mm Hg. He was alert and oriented to person, place, and time. In addition, he had no nuchal rigidity or neurologic deficit, and the rest of the systemic examination was within normal limits. On ophthalmologic examination, decreased eye movements with relative afferent pupillary defect due to optic nerve involvement was observed on the right side. The fundus examination revealed central retinal artery occlusion in the right eye. Furthermore, the examination of the nasal cavity revealed thick mucopurulent discharge in both of the middle meatuses. 

**Figure 1:**
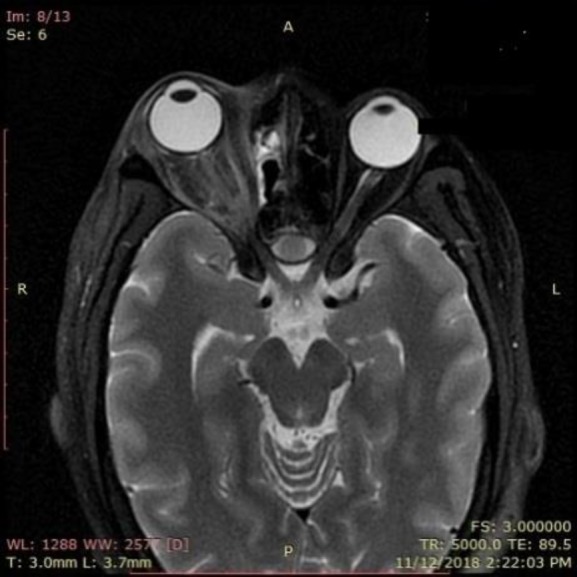
Contrast-enhanced magnetic resonance image showing fungal rhinosinusitis, along with proptosis of the right eye

Laboratory findings included a total leucocyte count of 11,500 per mm^3^ and mild neutrophilia (8,650 per mm^3^) with lymphocytes, eosinophils, monocytes, and basophils being in the normal range, as well as nonreactive results for HIV, hepatitis B virus, and hepatitis C virus. In addition, proteinuria and glycosuria in the urine, random blood sugar of 238 mg/dl, and hemoglobin A1C of 14.2% indicated uncontrolled sugar metabolism. 

Mucosal biopsy from the middle meatus was sent to the microbiology laboratory for fungal examination. On KOH wet mount, irregularly branched septate fungal hyphae were seen in the sample. Contrast-enhanced magnetic resonance imaging showed the features of ethmoidal sinusitis with intracranial extension across the lamina papyracea causing orbital cellulitis and ischemic optic neuropathy on the right side. Based on the above findings, the patient was diagnosed with type II diabetes mellitus with invasive fungal rhinosinusitis leading to orbital cellulitis.

The patient was started on insulin glargine injection (8 units) at bedtime, human regular insulin injection (6 units) three times a day, and clindamycin injection (600 mg) three times a day; however, no antifungals were prescribed. After the implementation of surgical debridement, the tissue sample was sent to the microbiology laboratory for the identification of fungal elements by wet mount and culture methods. The KOH wet mount showed irregularly swollen, branched septate fungal hyphae (Figure 1). 

In addition, the primary culture of the patient′s sample was performed on Sabouraud dextrose agar (SDA) and SDA with gentamycin and cycloheximide at two incubation temperatures of 37°C and 25°C. Four days after incubation, a dark brown mold was seen on the obverse side with nondiffusible black pigment on the reverse side of the growth (Figure 3). Lactophenol cotton blue staining from growth showed irregularly branched hyphae with spores having three septations (Figure 4). Based on the above micro-morphological findings, the diagnosis of rhino-orbital cellulitis due to *C. lunata* was established. Further confirmation was achieved by performing matrix-assisted laser desorption ionization time-of-flight mass spectrometry (Bruker Daltonics, Germany) which also led to the identification of the fungus as *C. lunata.*

**Figure 2:**
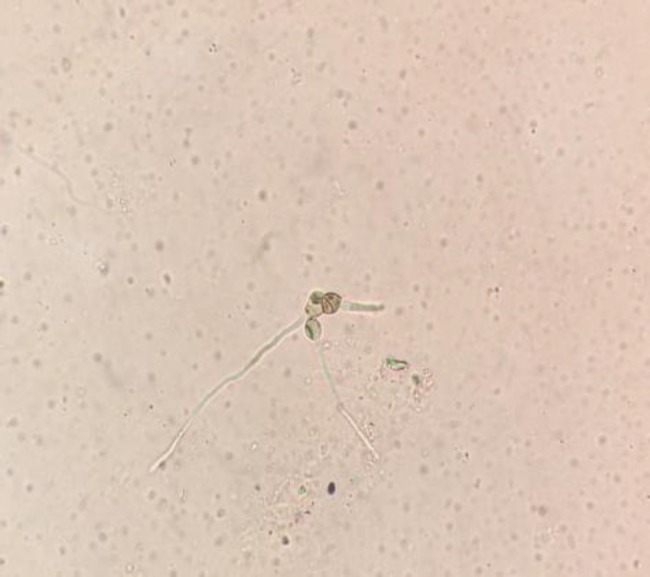
Direct wet mount of tissue sample showing irregularly branched hyphae with sporulation

Extensive surgical debridement was carried out, and the patient was started on intravenous (IV) voriconazole (6 mg/kg) every 12 h for 2 days, followed by 4 mg/kg IV every 12 h for 7 days [13]. Itraconazole (200 mg) was also added twice a day for 4 weeks. For the correction of the underlying condition, the patient was subjected to glargine injection (8 units) at bedtime and human regular insulin injection (6 units) three times a day. In the maintenance phase, itraconazole (200 mg) and subcutaneous glargine (10 units) were given at night for 4 weeks. The patient was followed up for 4 weeks. Blood sugar was well controlled, and nasal symptoms were absent; however, the loss of vision in the right eye was irreversible.

**Figure 3:**
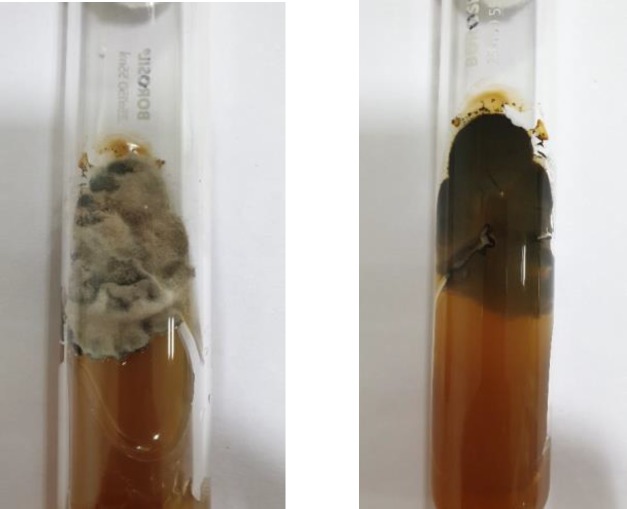
Culture on Sabouraud dextrose agar showing growth of *Curvularia* greyish brown colonies with pigment on the reverse side

**Figure 4:**
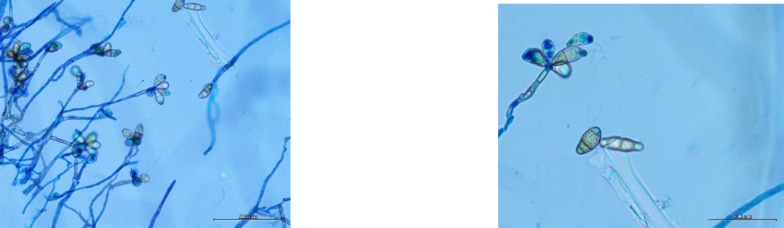
Lactophenol cotton blue wet mount of slide culture showing conidia of *Curvularia lunata* having three septae

## Discussion

Orbital cellulitis is defined as an infection in the structures situated posterior to the orbital septum. This condition mostly occurs as the result of infection spread from adjacent anatomical structures like paranasal sinuses. However, it may also occur either after trauma to the eye or due to hematogenous spread following bacteremia. This infection can lead to severe complications, such as a permanent loss of vision in the eye. Furthermore, this disease is more common in children than in adults [5].

A growing frequency of orbital cellulitis following community-acquired methicillin-resistant *Staphylococcus aureus* has been reported in the United States [6-8]. The most common etiological agents of orbital cellulitis are mixed bacterial infections, including both Gram-positive and Gram-negative bacteria. Among fungal etiological agents, *Aspergillus* and *Mucor* are the most common causative agents. Very few case reports regarding fungal orbital cellulitis due to dematiaceous fungi like *Alternaria,* *Bipolaris,* and *Curvularia* have been reported in the literature.

Early and prompt diagnosis of fungal orbital cellulitis is a matter of vital importance since this condition accounts for very high mortality in immunocompromised individuals, such as diabetic patients. In diabetic patients, the hyperglycemic state of the body favors fungal proliferation and decreases chemotaxis and phagocytic activity, thereby permitting the opportunistic fungi to thrive in an acid-rich environment [10-12]. Zygomycosis group of fungi, particularly *Mucor,* is more common in diabetic patients. There are several case reports in the literature regarding rhino cerebral mucormycosis in diabetic patients [9-12]. In addition, there is an increasing number of endophthalmitis and sinusitis cases caused by dematiaceous fungi. *Curvularia* has been reported in diabetic patients in recent studies; however, the case reports are very few in number [2-4, 13]. Increasing reports of dematiaceous fungi in immunocompromised individuals emphasize the importance of early and prompt diagnosis of such cases since the treatment for each group (i.e., dematiaceous and aseptate fungi) is entirely different.

Dematiaceous fungi are identified based on dark color colonies with dark reverse and pigmented multicellular spores on culture. In our case, sporulating structures with irregularly branched dark hyphae were seen in the direct wet mount examination of the intraoperative tissue sample (Figure 2). Based on the rapidly growing dark olive to brown colonies with dark reverse sympodial geniculate conidiation, *C. lunata* was detected as the etiological agent of the clinical condition in our patient. Conidia were large containing four cells with the central cell having a curved appearance. Conidia differ from *Bipolaris* which is a common differential diagnosis. Conidia has darker central cells than the terminal cells, thinner cell wall, narrower septations, and a distinct curve of the central cell [15, 16]

The outcome in patients with invasive fungal rhinosinusitis depends on the immunity of the host. Immunocompromised patients have poor outcomes because of altered immune mechanisms. The standard treatment of invasive fungal sinusitis is the extensive surgical debridement of the infectious source, along with systemic antifungal therapy with itraconazole and voriconazole [14]. Immunocompromised hosts also need the correction of the underlying problem [17]. 

## Conclusion

*Curvularia *infection is a very rare cause of invasive fungal rhinosinusitis in immunocompromised individuals as compared to the *Zygomycetes* group of fungi. Management of the aforementioned fungal groups is entirely different. It is important to emphasize that the possibility of emerging fungal pathogens should always be kept in mind whenever such clinical cases are encountered.
